# Supplementary material for: Enhancing restoration success of rare plants in an arid-tropical climate through water-saving technologies: a case study of Scalesia affinis ssp. brachyloba in the Galapagos Islands
Source: PeerJ. 2023 Dec 6;11:e16367. doi: 10.7717/peerj.16367 (PMC10710167; doi:10.7717/peerj.16367)
Supplement: Figure S1 — Lower values of SPA indicate younger plants, while higher older ones. In dots: raw binary data of dead and alive plants. [file peerj-11-16367-s003.pdf]

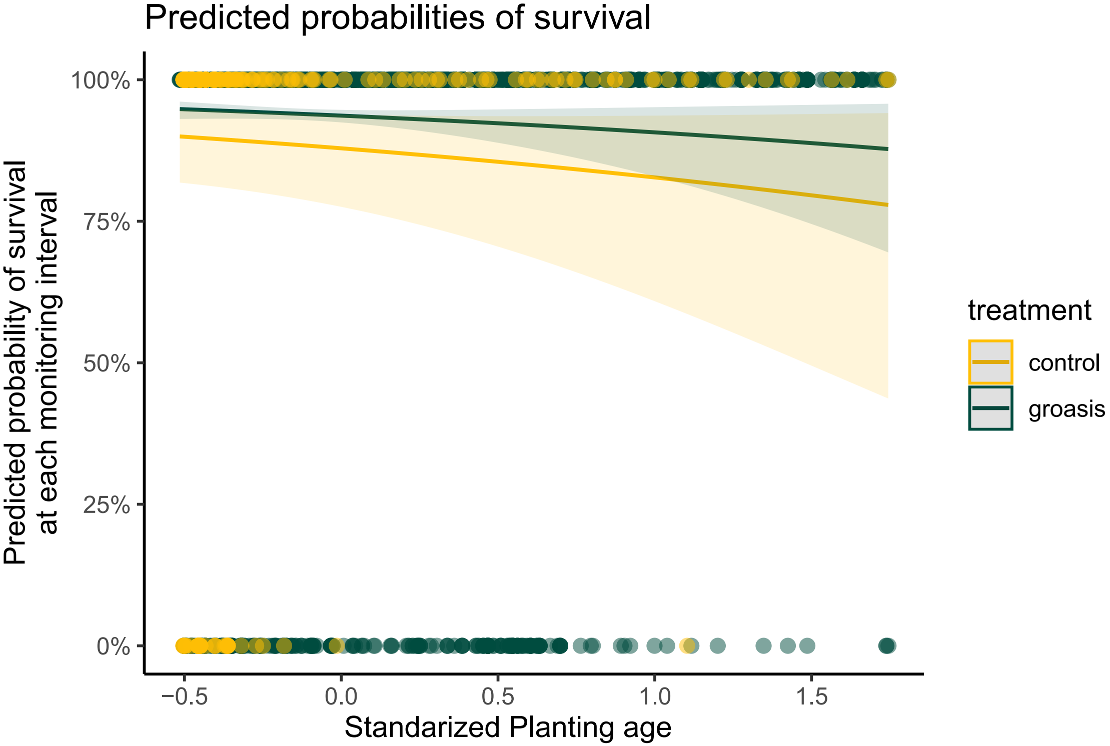

Supplemental Figure 1. Predicted survival as function of standardized plant age (SPA). Lower values of SPA indicate younger plants, while higher older ones. In dots: raw binary data of dead and alive plants.
