# Supplementary material for: Enhancing restoration success of rare plants in an arid-tropical climate through water-saving technologies: a case study of Scalesia affinis ssp. brachyloba in the Galapagos Islands
Source: PeerJ. 2023 Dec 6;11:e16367. doi: 10.7717/peerj.16367 (PMC10710167; doi:10.7717/peerj.16367)
Supplement: Figure S2 — (Upper graph) Estimated weekly mortality rate for Scalesia affinis ssp. brachyloba plantings on Santa Cruz Island; (Mid) Weekly maximum temperature (pink values = equal or lower than 27 °C, blue values = higher than 27 °C); (Lower graph) Weekly total precipitation in millimeters. [file peerj-11-16367-s004.pdf]

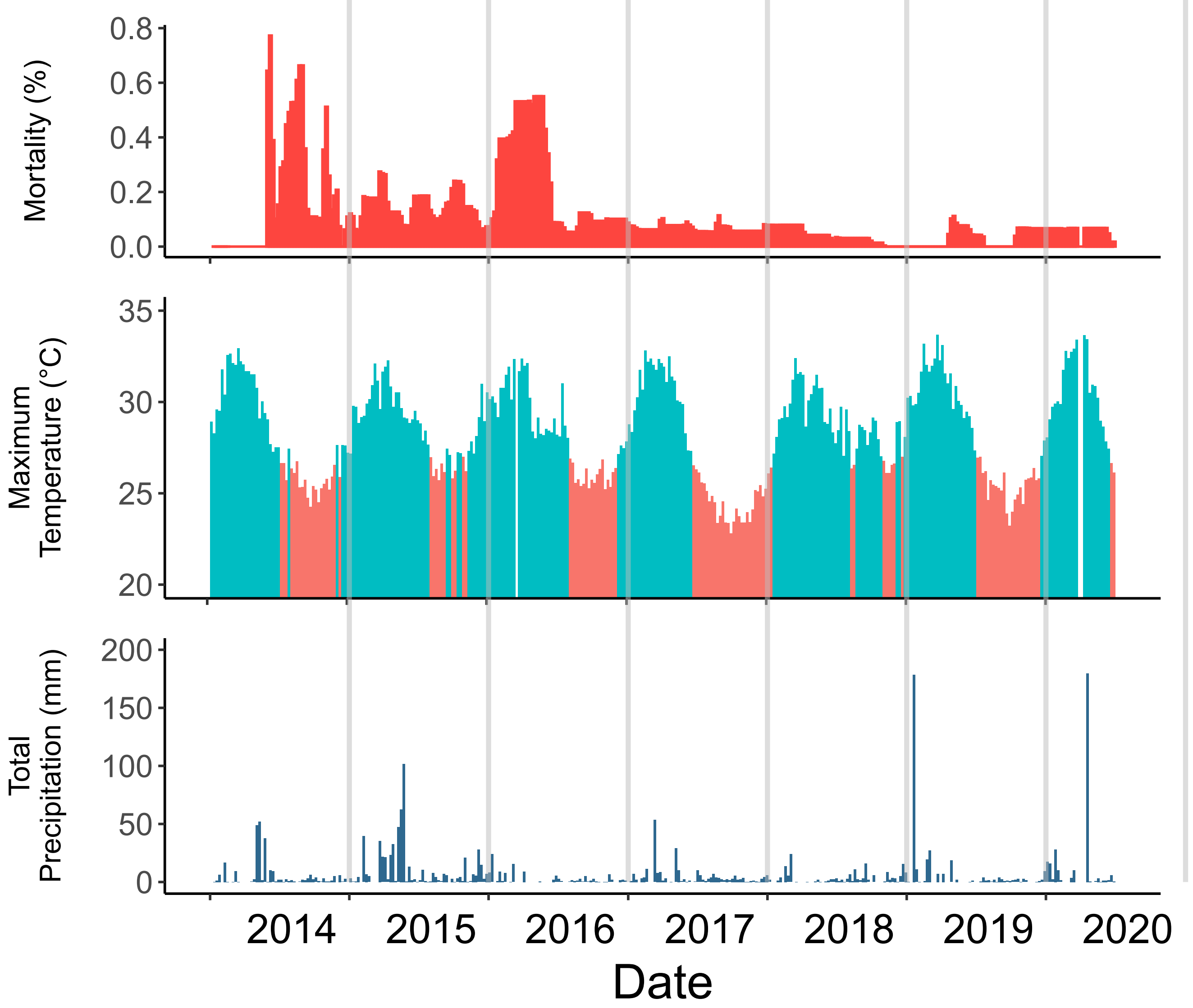

Supplemental Figure 2. Summary of weekly data of the study. (Upper graph) Estimated weekly mortality rate for *Scalesia affinis* ssp. *brachyloba* plantings on Santa Cruz Island; (Mid) Weekly maximum temperature (pink values = equal or lower than 27°C, blue values = higher than 27° C); (Lower graph) Weekly total precipitation in millimetres.
